# Perceptions of the general public and physicians regarding open disclosure in Korea: a qualitative study

**DOI:** 10.1186/s12910-016-0134-0

**Published:** 2016-08-20

**Authors:** Minsu Ock, Hyun Joo Kim, Min-Woo Jo, Sang-il Lee

**Affiliations:** 1 Department of Preventive Medicine, University of Ulsan College of Medicine, Seoul, South Korea; 2 Department of Nursing Science, Shinsung University, Dangjin, South Korea

**Keywords:** Patient safety, Open disclosure, Focus group discussion, In-depth interview

## Abstract

**Background:**

Experience with open disclosure and its study are restricted to certain western countries. In addition, there are concerns that open disclosure may be less suitable in non-western countries. The present study explored and compared the in-depth perceptions of the general public and physicians regarding open disclosure in Korea.

**Methods:**

We applied the COREQ (Consolidated Criteria for Reporting Qualitative Research) checklist to this qualitative study. We conducted 20 in-depth interviews and four focus group discussions with 16 physicians and 18 members of the general public. In-depth interviews and focus group discussions were performed according to semi-structured guidelines developed according to a systematic review of open disclosure. We conducted a directed content analysis by analyzing the verbatim transcripts and field notes in accordance with the predetermined guidelines.

**Results:**

Open disclosure perceptions were summarized in terms of the “five Ws and one H” (who, what, where, when, why, and how). All physician and general public participants acknowledged the normative justifiability of open disclosure. The participants mostly agreed on the known effects of open disclosure, but the physicians had negative opinions on its expected effects, such as decreased intention of the general public to file lawsuits and increased credibility of medical professionals. Generally, the participants thought that open disclosure is required for medical errors causing major harm. However, the physicians and general public had conflicting opinions on the need for open disclosure of near misses. Most physicians did not know how to conduct open disclosure and some physicians had bad experiences due to inappropriate or incomplete open disclosure.

**Conclusion:**

Physicians and the general public in Korea acknowledge the need for open disclosure. Guidelines according to the type of patient safety incident are required to encourage physicians to more readily conduct open disclosure. Furthermore, hospitals need to consider organizing a dedicated team and hiring experts for open disclosure.

**Electronic supplementary material:**

The online version of this article (doi:10.1186/s12910-016-0134-0) contains supplementary material, which is available to authorized users.

## Background

An understanding of the perceptions of medical professionals and the general public, including patients, regarding medical error is required to improve patient safety [1, 2]. Furthermore, medical professionals and the general public should discuss patient safety incidents caused by health care delivery and develop measures to prevent and manage them together [3]. However, there may be gaps between the perceptions of medical professionals and the general public regarding patient safety incidents, such as the causes of patient safety incidents and their prevention. In particular, when patient safety incidents occur, there may be a marked discrepancy between medical professionals and patients regarding the communication of the incident [4]. Patients and their caregivers want to know what has occurred, why it occurred, and how it will be handled. However, medical professionals are reluctant to openly communicate with patients and their caregivers after such incidents [2, 5]. These gaps in communication could diminish patient trust in medical professionals and patient satisfaction and may increase the likelihood of medical lawsuits [6, 7].

To promote the communication of patient safety incidents to patients and their caregivers, open disclosure programs and policies are being adopted in several countries [8–11]. The definition of open disclosure may differ among researchers and institutes, but key components of open disclosure usually include “an acknowledgement; an expression of regret or an apology; an investigation into the incident; providing a factual explanation of what happened and explaining the steps being taken to manage the incident and prevent recurrence” [12]. The known benefits of open disclosure include reduced intention of the general public to sue and punish medical professionals, increased trust in medical professionals, increased intention of patients to revisit and recommend hospitals or physicians, improved quality of care scores, and ameliorated feelings of guilt of medical professionals [13].

However, the experience with open disclosure is restricted to certain western countries [12]. Furthermore, there are concerns that open disclosure may be less suitable in non-western countries [14]. Therefore, in this qualitative study, we explored and compared the in-depth perceptions of the general public and physicians regarding open disclosure in Korea. In the case of Korea, open disclosure policies have not yet been adopted, but there is growing interest in patient safety including open disclosure due to the introduction of a new patient safety act that requires the establishment of a patient safety reporting system at a national level in 2016 [15]. The key findings from this qualitative study will help in the introduction of open disclosure programs and policies in non-western countries such as Korea.

## Methods

We performed 20 in-depth interviews (IDIs) and four focus group discussions (FGDs) to explore and compare the in-depth perceptions of study participants regarding open disclosure. A total of 34 individuals participated in this study (Tables 1 and 2). We thought that data saturation would be achieved with this sample size [16]. We applied the COREQ (Consolidated Criteria for Reporting Qualitative Research) checklist to this qualitative study as much as possible [17].

**Table 1:** Characteristics of the participants in the in-depth interviews

N	Sex	Physician/public	Age group
IDI-1	M	Physician	30s
IDI-2	M	Physician	30s
IDI-3	M	Physician	30s
IDI-4	M	Physician	30s
IDI-5	M	Physician	30s
IDI-6	M	Physician	30s
IDI-7	F	Physician	30s
IDI-8	F	Physician	30s
IDI-9	F	Physician	30s
IDI-10	M	Physician	30s
IDI-11	F	Public	30s
IDI-12	F	Public	30s
IDI-13	M	Public	40s
IDI-14	M	Public	30s
IDI-15	M	Public	40s
IDI-16	M	Public	40s
IDI-17	F	Public	30s
IDI-18	F	Public	30s
IDI-19	F	Public	40s
IDI-20	M	Public	30s

*N* number, *IDI* in-depth interview, *M* male, *F* female

**Table 2:** Characteristics of the participants in the focus group discussions

N	Sex	Physician/public	Age group	Mixed group
FGD-1	F	Physician	30s	First
FGD-2	M	Physician	30s	First
FGD-3	F	Physician	30s	First
FGD-4	F	Physician	40s	Did not attend
FGD-5	M	Physician	40s	Second
FGD-6	F	Physician	30s	Second
FGD-7	F	Public	40s	First
FGD-8	F	Public	50s	First
FGD-9	M	Public	40s	First
FGD-10	M	Public	30s	First
FGD-11	M	Public	50s	Second
FGD-12	M	Public	50s	Second
FGD-13	F	Public	30s	Second
FGD-14	F	Public	40s	Second

*N* number, *FGD* focus group discussion, *M* male, *F* female

### Organization of the research team

The research team consisted of four members. Three of the team members (MO, MWJ, and SIL) had previously participated in other studies into patient safety. Two of the team members (MO and HJK) had repeated experience in conducting and publishing qualitative studies.

### In-depth interviews

IDIs were conducted with 10 members of the general public and 10 physicians. We operationally defined the general public as persons with no license or certificate in the medical area. We recruited members of the general public with a college diploma in their 30s or 40s, considering comparability with the physician participants. At the beginning of recruitment, IDI participants were purposively selected from personal contacts of the authors and we additionally recruited participants recommended by the early IDI participants. Therefore, in terms of reflexivity, we cannot exclude the possibility that the IDI participants were not completely open with the researchers and tried to ingratiate themselves with the interviewer. There was no one who refused to participate in and dropped out. IDIs were conducted in a quiet room in a hospital from April 2015 to June 2015. One author (MO) interviewed the participants according to the semi-structured guidelines. The interview lasted about 1 h on an average and was audio-recorded.

### Focus group discussions

We conducted four FGDs. The participants of the first FGD were the general public, whereas physicians participated in the second FGD. The participants of the third and fourth FGDs were a mixture of the general public and the physicians from the first and second FGDs. Six to eight participants were involved in each group. We selected physicians with experience of patient safety incidents, as in the IDIs, to participate in the FGDs, but Gallup Korea recruited the general public participants of the FGDs by selecting individuals who were interested in medical accidents and aged in their 30s to 50s. Therefore, we judged that the general public participants of the FGDs could talk freely in terms of reflexivity, although there was a possibility that the physician participants of FGDs might not have felt open to frankly discuss sensitive issues such as experiences with patient safety incidents. No physicians refused to participate in the FGDs. However, we cannot determine the number and characteristics of the general public who refused to participate in the FGDs because Gallup Korea recruited these participants among the panel members established by Gallup Korea. The FGDs were conducted in a one-way mirror room at Gallup Korea. One author (MO) and one professional interviewer from Gallup Korea led the discussion according to the semi-structured guidelines, similar to the IDIs. All FGDs were conducted in May 2015. Each FGD lasted about 2.5 h on average and was audio- and video-recorded.

### Development and construction of guidelines

We developed the semi-structured guidelines for IDIs and FGDs based on a systematic review of open disclosure [13]. The guidelines were constructed to comprehensively discuss open disclosure issues according to the principle of the “five Ws and one H” (who, what, where, when, why, and how; Additional file 1). In particular, we introduced five hypothetical cases to address various types of patient safety incidents requiring open disclosure: (1) inapparent medical error causing minor harm, (2) inapparent medical error causing severe harm, (3) apparent medical error causing minor harm, (4) apparent medical error causing severe harm, and (5) failure of open disclosure in the case of apparent medical error causing severe harm. By assuming no harm in our third hypothetical case, we determined the participants’ perceptions regarding a near miss. We expected that the hypothetical cases would help participants to readily express their opinions on open disclosure, but we did not just focus on these hypothetical cases.

### A definition of terms

Before conducting the IDIs and FGDs, we clarified a definition of terms related to patient safety to the participants. We defined patient safety incident as “an event or circumstance that could have resulted, or did result, in unnecessary harm to a patient and medical error as “a failure to carry out a planned action as intended or application of an incorrect plan”, in accordance with “Conceptual Framework for the International Classification for Patient Safety” from the World Health Organization [18]. We also defined adverse event as “an injury resulting from a medical intervention, or in other words, it is not due to the underlying condition of the patient” and near miss as “medical errors that resulted in no harm”, in accordance with the Institute of Medicine [19]. The term of open disclosure were from the Australian open disclosure framework [9] and open disclosure was defined as series of process as follows: “When a patient safety incident occurs, medical professionals preemptively explain the incident to the patients and their caregivers, express sympathy and regret for the incident, deliver apology and compensation appropriately if needed, and promise to prevent recurrence.”

### Analysis

The audio recordings from the IDIs and FGDs were transcribed verbatim. Transcripts were not returned to participants for comment or correction. We applied a directed content analysis by analyzing the verbatim transcripts and field notes in accordance with the predetermined guidelines of IDIs and FGDs [20]. One author (MO) first read the transcriptions and field notes repeatedly and coded them according to the categories. Another author (HJK) reconfirmed the results of the analysis. Data saturation was confirmed by two coders when no additional codes were identified. If there were disagreements, two coders conducted a reiterative analysis together and reached an agreement. The research team also examined the codes and their categories. We used NVivo 10 software for the analysis [21]. We did not get feedback from participants on the results of the analysis.

## Results

A total of 34 individuals participated in the IDIs (*n* = 20; Table 1) and FGDs (*n* = 14; Table 2). Analyses of the transcriptions identified 474 codes and the codes were categorized into nine groups according to the “five Ws and one H”. The structure of the analysis results and the main content is shown in Table 3. Further details are provided below.

**Table 3:** Structure of the analysis results and the main content

5W1H of open disclosure	Category	Main content
Why	Why should medical professionals conduct open disclosure?	• Open disclosure is an imperative, especially for the healthcare field because it deals with matters affecting life and death. • The well-known benefits of open disclosure, such as reduced number of medical lawsuits and improved level of patient trust in physicians, were generally accepted by participants.
When	When should open disclosure take place?	• Physicians and the general public agreed on the need for open disclosure in the event of medical errors causing severe injuries. • Most physicians expressed marked negativity toward open disclosure of near misses, but most participants from the general public group demanded open disclosure of near misses for the patient’s future reference and to prevent recurrence.
	When should open disclosure be initiated?	• Participants from the general public group wanted open disclosure to take place as promptly as possible.
What	What should be delivered through open disclosure?	• Incident description and sympathy expression were considered essential elements of open disclosure. • The general public mainly argued that if harm occurs due to medical error, the apology should come before compensation and will bring about emotional relief.
Who	Who should practice open disclosure?	• Physicians thought that implementation of open disclosure itself depends on the superior involved, that is, the professor in charge of the department.
	Who is subject to open disclosure?	• Many participants believed that not only patients, but also caregivers are subject to open disclosure.
Where	Where should open disclosure take place?	• All physicians insisted on a separate place, for instance, a quiet counseling room, as a venue for open disclosure.
How	How should open disclosure be carried out?	• Physicians did not know how to conduct open disclosure. • The general public felt offended by inadequate apologies and consequently felt neglected in other medical areas as well.
	How should open disclosure be promoted?	• Most physicians acknowledged the importance of open disclosure education. • Not only most physicians, but also considerable numbers of participants from the general public showed support for improving the medical environment, that is, the organizational culture of hospitals. • Several participants requested establishment of a system after the introduction of open disclosure guidelines. • Many agreed on the purpose and need for an apology law to facilitate open disclosure.

*5W1H* five Ws and one H

### Why should medical professionals conduct open disclosure?

All of the participants in this study, both the physicians and the general public, acknowledged the need for open disclosure. In essence, many suggested that open disclosure is required in its own right, regardless of its impacts or benefits. That is, they felt that open disclosure is a physician’s moral responsibility and that patients’ and their caregivers’ have a right to know this information. Therefore, physicians with a clear conscience would conduct open disclosure and those who neglect to do so could never be considered honorable. Moreover, participants considered that open disclosure is an imperative, especially for the healthcare field because it deals with matters affecting life and death.

- *The need for open disclosure*
  *Interviewer: Then, do you think open disclosure should be put into practice in that specific circumstance mentioned before?*
  *In-Depth 7 (Physician): Yeah, it should.*
  *Interviewer: Why so? For its own benefits? Or do you have other reasons in mind?*
  *In-Depth 7 (Physician): Of course, such benefits can be considered. But, rather, first the patient, the caregiver or the patient, should understand the situation and what s/he is getting. Even if things go badly, the person in question has to know what is precisely being done to himself/herself. In a sense, it is a moral responsibility.*

The well-known benefits of open disclosure, such as reduced number of medical lawsuits, improved level of patient trust in physicians, increased willingness to revisit the physician/hospital, higher patient satisfaction, and lessened physician guilt were generally accepted by participants. However, some were skeptical. In particular, physicians were cynical of the effect of open disclosure on reducing lawsuits; they forecasted that the fall in the number of medical lawsuits would be hindered by Korean laws and the healthcare milieu.

- *Skeptical stance of physicians on the effect of open disclosure on reducing medical lawsuits*
  *Interviewer 2: It may be a foreign case, but studies do show reduced numbers in medical lawsuits upon adoption of open disclosure. What are your thoughts on that? I am talking about a decline in overall filings.*
  *Focus 6 (Physician): Well, if that’s what we’re after, we cannot be more mistaken. The number of attorneys are on the rise, you know.*
  *Focus 3 (Physician): Brokers barge into the ICU.*
  *Focus 6 (Physician): I have a friend who’s a lawyer and s/he says they are increasingly facing the same thing....*
  *Interviewer 2: But the number of attorneys is higher in the States, isn’t it?*
  *…*
  *Focus 6 (Physician): It worked and the numbers dropped. Really? Haven’t read any papers on it.*

### When should open disclosure take place?

In deciding when to conduct open disclosure, there was discordance among participants according to the characteristics of the patient safety incident. First of all, physicians and the general public agreed on the need for open disclosure in the event of medical errors that cause severe injuries. A medical error leading to severe harm is an indisputable event. Therefore, many suggested that such a trait leaves physicians no choice but to conduct open disclosure. Meanwhile, others pointed out the possibility of concealing, rather than disclosing, medical errors resulting in severe injuries.

- *Need for open disclosure of medical errors causing severe harm*
  *Interviewer 2: In this case (fifth hypothetical case), do you think open disclosure should be implemented?*
  *Focus 3 (Physician): Yes, I do.*
  *Focus 2 (Physician): Sure. I feel sorry, emotionally and not only that… I feel very…*
  *…*
  *Interviewer 1: How about others?*
  *Focus 10 (Layperson): I think open disclosure should be implemented, too. A physician’s malpractice resulted in death of a patient, so I’m guessing the physician should be feeling quite guilty. Open disclosure is definitely needed.*

On the other hand, comparative comments were made for medical errors that resulted in minor harm. For such errors, open disclosure could be practiced without much stress, so the likelihood of implementation is high. However, one physician mentioned that s/he would inform the hospital patient safety reporting system of medical errors resulting in minor harm but would not practice open disclosure with the patient and the caregivers; another participant voiced concern over such a comment, stressing that it will decrease the patient’s trust in medical staff and reduce their willingness to revisit the hospital. In particular, physicians mostly assumed that patients would not want to know about medical errors causing minor problems.

- *Pessimistic perspectives of physicians on the need for open disclosure of apparent medical errors causing minor harm*
  *Focus 6 (Physician): I suppose medical errors causing minor harm will be even more problematic.*
  *Interviewer 2: Because patients will know nothing of it if the physician keeps quiet?*
  *Interviewer 1: You know you should say something, so you are facing a dilemma as a doctor?*
  *Focus 3 (Physician): Hmmm. I’d rather not say. This is a matter of preference, I think. The patient might not feel the need either. Telling the truth is the right thing to do, but since nothing really happened, I guess doctors would be inclined not to do so.*
  *Focus 6 (Physician): If I were a patient, I’d rather not know.*

Physicians were markedly against carrying out open disclosure for near misses. They were skeptical of the need for open disclosure and determined that it would increase neither the patients’ nor the caregivers’ trust in physicians. However, most participants from the general public and a few physicians demanded open disclosure for near misses for the future reference of patients and to prevent recurrence.

- *Perspective of a participant from the general public group on the need for open disclosure for near misses*
  *In-Depth 19 (Layperson): I needed some time to think. Would open disclosure be necessary when nothing actually happened? Would it be okay not to say anything because nothing happened?*
  *Interviewer: Wouldn’t sweeping it under the rug make things easier for both the patient and the doctor?*
  *In-Depth 19 (Layperson): Actually, it did come across and it’s become a real hang-up for me…*
  *Interviewer: But you support open disclosure in spite of that because…?*
  *In-Depth 19 (Layperson): Because I have been in that situation. It occurred to me that I should be aware of how it could seed a dispute. Plus, if I were to appeal a malpractice later on, I’d better learn the facts at least. I may or may not have complications, but if certain symptoms do occur and say it causes problems… I’ve thought it over and over and it led me to the conclusion that honesty is the best policy.*

Finally, almost all physicians had negative views on practicing open disclosure of inapparent medical errors. Many physicians found it difficult to implement open disclosure in vague situations and pointed out how hard it is to manage such situations. On the other hand, a number of participants from the general public group set forth their opinions on the need for expressing regret and apologizing even for inapparent medical errors. Furthermore, there were demands for compensation for the harm done to the patient, irrespective of the characteristics of the medical error.

- *Need for an apology for inapparent medical errors*
  *Focus 14 (Layperson): Whether the doctor admits it or not, an apology is a must. The mind and the body are not separate, you know. The mind is actually connected to the body and directly affects it, so the patient ought to have been extremely confused and troubled. I mean, the least you could do is to admit that they’re liable and apologize. Say, “I’ve tried my best as your doctor and I am sorry for the unintended consequences.”*

In particular, certain physician participants admitted that there is a gap between the perspectives of the general public and physicians about which particular adverse events are subject to open disclosure and whether a medical error has occurred or not. Moreover, they required a proactive approach when dealing with open disclosure of all patient safety incidents.

- *Differences between the perspectives of physicians and the general public on patient safety incidents*
  *Focus 5 (Physician): To bring our attention back to this issue, as you have seen here, the major premise is that a patient safety incident had occurred. But the thing is… the standpoints of seeing this as a patient safety incident vary. Doctors do not consider it such an incident.*

### What should be delivered through open disclosure?

The four elements of open disclosure—providing explanation, showing sympathy and guaranteeing future investigation, offering an apology, and promising adequate amount of compensation and effort to prevent recurrence—were reviewed in this qualitative study. Firstly, most participants, including several physicians, and particularly the bulk of participants belonging to the general public group supported the early stages of open disclosure, such as providing explanation, showing sympathy, and guaranteeing future investigation. The emphasis was on the importance of reassuring the patient, providing emotional comfort, and promising investigation of the matter when a patient safety incident occurred. Particularly, incident description and sympathy expression were considered essential elements of open disclosure. Thus, omitting them would tarnish the meaning of the other elements. In addition, there were opinions on the need for an explanation despite the ambiguity of the medical error.

- *Need for incident explanations in open disclosure*
  *In-Depth 8 (Physician): Whatever the case, you should give an explanation to the caregivers. On why you did it and how things can go wrong.*
  *Interviewer: And what makes you think you should inform them?*
  *In-Depth 8 (Physician): Well, isn’t it natural for them to want to know? All of a sudden, a patient dies after a surgery. Sure, the odds of death from surgical complications, one in a million and whatnot, are well known. But they probably wouldn’t have imagined anything like it happening in their own life, not in a million years.*
  *Interviewer: Then, it’s for appropriateness? Because you ought to or is it out of some sort of ethical conscience?*
  *In-Depth 8 (Physician): It’s designated in medical ethics, and aren’t we mandated to inform the caregivers because it’s just right?*

Secondly, many participants showed emotional support for an apology, which is the second element of open disclosure. The general public mainly argued that if harm occurs due to medical error, an apology should come before compensation and will bring about emotional relief. However, for fatal accidents, some participants from the general public group mentioned concerns regarding the acceptance of apologies because their power was limited.

- *Importance of delivering an apology in open disclosure*
  *Focus 7 (Layperson): The main target of open disclosure seems to be obtaining sincere apologies from physicians. Apart from money, as a human being, one-on-one… When a patient is harmed or dies, we want a wholehearted apology. Medical disputes come later. Money and whatnot comes second.*
  *Interviewer 2: I’d like to speak on behalf of physicians because they found that “it all comes down to money”.*
  *Focus 7 (Layperson): Not all people are like that. A good tongue is a good weapon, you know. With a heartfelt “sorry”…*
  *Focus 1 (Physician): But let’s say your father had passed away. He was the one providing for the family. So, now you are all of a sudden out of money. Will an apology do? Your mother died so now you need to hire a housekeeper. Will it do? It goes the same for a child too. A kid does not even support the family, but most people seem to consider financial compensation a sincere way of apologizing.*
  *Focus 10 (Layperson): Money is not the best policy.*
  *Focus 7 (Layperson): Money is not a cure-all. Father’s gone. The only source of family income is gone now and no sincere apology or whatsoever will replace him. But if doctors take responsibility and apologize for my loss, I would take it on my own shoulders even when I have to earn a living for myself. Because I accepted the apology.*

In addition, not only the physicians, but also the general public insisted on an apology only when a medical error occurred. However, there were few opinions from the general public on how they would emotionally prefer an apology, with some admitting that it is rationally unnecessary. Most participants distinguished between sympathy and an apology but had trouble defining them. One of the participants who belonged to the general public group suggested that an apology cannot be considered an admission of medical error or negligence, depending on the expressions used and underlying context.

- *Apology not regarded as admitting negligence*
  *In-Depth 19 (Layperson): When such a case arises, it’s saying sorry for what’s happened and any wrongdoing possibly caused by the medical staff, for example. And it doesn’t necessarily mean you are admitting negligence. At first, when people are in a fury or are upset, they may take it as an admittance [of guilt]. But after they cool down and think about it, I don’t think they will take it that way. And, personally, I wouldn’t.*

Thirdly, most participants from both groups urged a guarantee of adequate compensation if the medical error was clear. When the medical error is not apparent, physicians need not promise compensation. Furthermore, some thought that an adequate amount of compensation for adverse events would prevent disputes. However, there were worries about how the ambiguity of the word “adequate” could cause difficulty in determining the amount of compensation.

- *Compensation unnecessary for inapparent medical errors*
  *In-Depth 12 (Layperson): I don’t see any need for compensation. If it’s not an error, an apology will do. Compensation is absolutely unnecessary and saying sorry for the inconvenience caused seems fine.*
  *Interviewer: Isn’t it unfair for the patients? They came to a hospital in the hopes of getting better, but end up with a longer hospital stay and additional treatment.*
  *In-Depth 12 (Layperson): Now that’s something we have to work on. “Due to this condition, this rare, probability of one or two percent, complication occurred. That is why you need further treatment and I am sorry for the inconveniences caused.” Good enough. You do not need to provide compensation.*

Fourthly, discordance between the perspectives of physicians and the general public became apparent in the fourth element of open disclosure, promising to prevent recurrence. Several physicians did not feel the need to promise prevention. Furthermore, similar to apologizing and assuring adequate compensation, they refused to accept the fourth element when the medical error is not apparent.

- *Skeptical perspectives of physicians on a promise to prevent recurrence*
  *Interviewer: Do you think promising to prevent recurrence is a different matter?*
  *In-Depth 2 (physician): I think education is a prerequisite if we were to jump into that. But in this situation where everyone’s growling at each other… I think it’s better not to…*
  *Interviewer: Even if it’s for future recurrence prevention?*
  *In-Depth 2 (physician): That’s a promise you have to make for yourself, not to the patient or the caregiver…*

However, many participants from the general public insisted on a promise of recurrence prevention to the patient and the caregiver, because they thought that prevention itself should be our ultimate aim. In addition, despite the ambiguity of the error, prevention was deemed important to identify the hidden factors behind the incident.

- *Need to promise recurrence prevention in ambiguous medical errors*
  *Interviewer: Then, how about assuring recurrence prevention?*
  *In-Depth 18 (Layperson): Well, this is a must, whatever the case.*
  *Interviewer: But you can only promise something when you know what you’ve done wrong, no? If the doctor seems clueless, would his/her apology sound sincere to you?*
  *In-Depth 18 (Layperson): What I’m wondering is… Is the doctor confident about his actions in this situation? Completely positive?*
  *Interview: S/he may or may not.*
  *In-Depth 18 (Layperson): Even if it’s not a detailed apology, I’m sure when doctors say how sorry they are for what happened and reassure [the patients] that they’ll make an effort to reduce possible complications, the patients will go back home feeling much better. No benefits whatsoever, but credibility will soar, I reckon.*

### How should open disclosure be carried out?

Generally, physicians were aware of the significance of successful implementation of open disclosure. However, they did not know how to carry it out. Furthermore, they shared their experiences on how inappropriate or imperfect open disclosure either provoked negative responses or turned out to be ineffective. They also seemed to have acquired certain coping skills, such as avoiding bringing up words or phrases that might trigger hostile reactions from patients. Meanwhile, some could not practice open disclosure even under compulsory circumstances.

- *Physicians’ styles in carrying out open disclosure*
  *Focus 6 (Physician): I don’t literally bring up the word “regrettable”, but I do it eventually.*
  *Interviewer 2: How do you express it without actually saying [the word]?*
  *Focus 3 (Physician): Like, “You won’t be able to go home, unfortunately”.*
  *Focus 6 (Physician): “We need further tests”.*
  *Interviewer 1: Without using the word “sorry”?*
  *Focus 3 (Physician): It’s a Korean thing that you don’t really need to put into words to…*
  *Focus 6 (Physician): “We need more tests”.*
  *Focus 3 (Physician): The biggest problem is when you’re about to discharge your patient after stitch removal, the last step of the surgery, the wound starts to open up. It’ll drive you crazy and what can you say to the patient? “Seems like you can’t go home today”.*
  *Focus 2 (Physician): That’s the Korean way of saying sorry.*
  *Focus 3 (Physician): You don’t really need to say it through words.*
  *Focus 5 (Physician): But surely there’s a gap between how doctors and patients feel.*

However, the general public felt offended by inadequate apologies and consequently felt neglected in other medical areas as well.

- *Offended by inadequate delivery of apology*
  *Focus 14 (Layperson): The apology did not make me feel any better. ‘Cause I knew how inattentive the head nurse at that floor was. I kept my eyes on that child 24/7, except for that 30 min, during which time the tragedy occurred. If I hadn’t come across it, who knows what would’ve happened? But that grumpy chief nurse just wouldn’t listen to me.*
  *Interviewer 1: Did she apologize?*
  *Focus 14 (Layperson): Technically, yes. Only because the director of the hospital told her to.*
  *Interviewer 1: What do you think about that?*
  *Focus 14 (Layperson): Completely fake. She kept on fussing about things so I had a word with the director of the hospital. I told him to fire the nurse. I mean, what is he gonna do if tragic accidents occur?*

In particular, most participants could not present a clear-cut means to deliver empathy and apology, the two elements of open disclosure, in a cordial way. However, they expected that sincerity would be expressed through the following: precise and detailed explanations, use of charts and data, and investing time in counseling. Additionally, one physician felt that more time spent with the patient reinforced the persuasiveness of his/her explanation. One participant from the general public group pointed out that a sincere apology can be intuitively distinguished from an apology that is feigned.

- *Ways to express sincerity during open disclosure*
  *Interviewer: Then, how does a person know that s/he’s cordially, honestly trying to express sympathy?*
  *In-Depth 1 (Physician): Hmmm… It’s tough.*
  *Interviewer: What can I do to make them understand and show how deeply I connect with them? Sounds pretty hard, I know…*
  *In-Depth 1 (Physician): You’re right. How should I put this… Nothing really comes to mind.*
  *Interviewer: Have you had any experiences like this?*
  *In-Depth 1 (Physician): As in having no one believing in what I have to say, even when I’m being honest?*
  *…*
  *In-Depth 1 (Physician): Truth be told, I think there’s no magic in doing it. Having conversations often can work too, I guess. We go on rounds several times a day, so it’s doable.*
  *Interviewer: Then, you’re saying frequent interactions are important for open disclosure?*
  *In-Depth 1 (Physician): Possibly. Of course, it’s not the number of contacts that’s important… Or, you know, we can talk while we look at charts or images and those things are good backups for conversations. It’ll be much better than face-to-face talks, no? I don’t know how I should put this…*

### How should open disclosure be promoted?

Measures to promote open disclosure can be classified into four categories: educating medical staff about open disclosure, improving the medical environment as a whole, introducing open disclosure guidelines, and reforming the legal framework. First of all, most physicians acknowledged the importance of open disclosure education. They confessed that they had never received training on doctor-patient communication, much less on open disclosure. Thus, from their own experience, physicians were afraid of how the patients and caregivers would react to the disclosure of a patient safety incident. In addition, their fear over miscommunications placed greater strain on the implementation of open disclosure.

- *Absence of open disclosure education*
  *Focus 3 (Physician): I have never learned (open disclosure). Can’t make facial expressions. Can’t come up with words to say…*
  *…*
  *Focus 1 (Physician): I have never seen anyone do it, so I don’t have a clue on how to do it.*
  *…*
  *Focus 1 (Physician): Not only that, but also IV fails, tapping fails, bone marrow (aspiration) fails in the ER… These days, I am affiliated to an online club of moms of pediatric cancer patients as an observer. They talk about all kinds of things there. One of the things that grabbed my attention was how moms were upset after repeated failures and no apologies from doctors. Moms were very upset. Failed procedures were making their kid suffer in pain, but doctors would smile and walk away. We can imagine why doctors would smile in such situation. They were embarrassed and that’s probably why they smiled awkwardly. Moms get furious because their babies are sick. But, in fact, we are extremely sorry, but we just don’t know how to express or convey it.*

Secondly, not only most physicians, but also a considerable number of participants from the general public showed support for improving the medical environment, that is, the patient safety culture of hospitals. In reality, even with hospital approval, it is not easy to perform open disclosure, and the hierarchical hospital culture that involves apprenticeship and the traditional “blame culture” was indicated as a factor further impeding open disclosure. One physician grimly forecasted the likelihood of practicing open disclosure in a setting where physicians are instructed by supervising physicians or consultants to not apologize.

- *Difficulties of open disclosure due to exclusive organizational culture*
  *Interviewer: Will open disclosure be possible in Korea?*
  *In-Depth 20 (Layperson): No.*
  *Interviewer: Why not?*
  *In-Depth 20 (Layperson): Systematic problems should be resolved to invigorate the practice of open disclosure, but internal relationships among doctors are just as important. I mentioned talking to the supervising doctor because I heard how the medical community resembles the military. If you are at arm’s length with someone, when you tell them “I’ll say it”, then they will show sympathy or not. However, in a strict environment, where they can sort of command you not to say, then it will be tough.*

Thirdly, several of the participants requested establishment of a system after the introduction of open disclosure guidelines because single-handed open disclosure is tough and prior preparation is needed. Some called for a dedicated team or experts responsible for open disclosure. However, others negatively viewed the effects of guidelines on the actual implementation of open disclosure, demanding an improvement in the overall medical environment.

- *Need to establish a system for open disclosure*
  *Interviewer: You mentioned that it’s a systematic problem (as to why open disclosure cannot be practiced), so what kind of system should there be?*
  *In-Depth 11 (Layperson): There should be a legal team or some sort of personnel to consult when such things happen. By the way, do physicians know how to manage certain situations when they come up?*
  *Interviewer: I’m not sure.*
  *In-Depth 11 (Layperson): They probably won’t, I guess. So, there should be expert team for that.*

Finally, participants were asked how they viewed the apology law and open disclosure law in light of the legal reform. Many agreed on the purpose and the need for an apology law to facilitate open disclosure. In other words, the apology law could dispel concerns among physicians who worry about having an apology used against them in court. Meanwhile, several others doubted the likelihood of the introduction of an apology law and its usefulness. Some physicians even suggested the possibility of an increase in medical lawsuits filed by patients informed about patient safety incidents that would have been overlooked without open disclosure. In addition, some participants from the general public expected an increased number of patient-doctor conflicts in Korea upon the introduction of open disclosure compared with the United States. One participant from the same group thought that empathy or apology expressed under the apology law would hardly be sincere. Moreover, several physicians predicted that open disclosure would be practiced only in incidents that are distinct to the patients or the caregivers.

- *A participant from the general public group supporting an apology law*
  *In-Depth 20 (Layperson): For now, it will definitely invigorate open disclosure. But, now that I think of it, patients might not consider open disclosure sincere in severe cases. It won’t feel like doctors are being 100 % frank. It’ll give the impression that they are trying to duck out of responsibility and say sorry at the same time, so I guess it won’t make patients any happier.*
  *Interviewer: Okay.*
  *In-Depth 20 (Layperson): Nevertheless, it’s a necessary evil. When such a system is nonexistent, these things will never be disclosed to begin with. It’s like a necessary evil.*

Participants expressed doubt about the adoption of an apology law because of the differences between the legal systems in the United States and Korea. However, despite the probability of weakening a patient’s ability to prove medical malpractice in court, most people from the general public were still in favor of an apology law. However, some insisted on imposing limitations on the starting time of open disclosure in order to enact an effective apology law. Therefore, they suggested protecting open disclosures that have been carried out within a certain time after the occurrence of the patient safety incident or when it is acknowledged by physicians.

- *Participants from the general public group supporting an apology law even when patient’s admissible evidence can be tainted*
  *Interviewer 2: How do you feel about the fact that an apology law could restrict the admissibility of patients’ statements?*
  *Focus 3 (Physician): It’s almost impossible to prove medical negligence, for your information.*
  *Focus 9 (Layperson): Are you saying that there’s favoritism toward physicians?*
  *Focus 1 (Physician): Statement itself is not accepted as evidence. Let’s say a doctor admitted negligence verbally. But it simply has no power in court.*
  *Interviewer 2: Not considered a confession… Are you really okay with this? It’s not easy to prove.*
  *Focus 7 (Layperson): If a doctor says, “there was an error in medication administration” in open disclosure, the statement cannot hold him/her liable, huh?*
  *Focus 3 (Physicians): Instead, you should present other data to prove erroneous drug administration.*
  *Focus 7 (Layperson): Conversations can easily be manipulated in situations where medical disputes arise or someone dies from malpractice. If you can mess with medical records, why not with words? The victims rightfully deserve the doctor’s apology. And, regarding the liability issue of doctor’s statements, I guess I’m okay with it.*

## Discussion

We conducted 20 IDIs and four FGDs with 16 physicians and 18 members of the general public to explore and compare their in-depth perceptions of open disclosure. We found specific findings under the various aspects of open disclosure in terms of the “five Ws and one H”. We also compared the differences and similarities between physicians’ and the general public’s perceptions of open disclosure. The key findings from this qualitative study will help to promote a more positive view of open disclosure and to develop open disclosure guideline policies in hospitals. In particular, this study will broaden the understanding of open disclosure in non-western countries because studies on open disclosure are often scarce in non-western countries.

### The need for open disclosure

Although there are concerns that open disclosure is less suited to non-western countries [4], all of the participants in this Korean study acknowledged the need for open disclosure. In particular, many suggested that open disclosure is required in its own right, regardless of its impact or benefits. They thought that open disclosure is an imperative for the healthcare field because it deals with matters of human life. Furthermore, open disclosure is valuable, in terms of not only patient safety, but also patient-centered care, emphasized by the Institute of Medicine as one of the goals for healthcare improvement [22]. We need to pay more attention to what is right and wrong based on the views of patients and their caregivers.

The well-known benefits of open disclosure, such as a reduced number of medical lawsuits, improved level of patient trust in physicians, increased willingness to revisit physicians/hospitals, higher patient satisfaction, and ameliorated guilt in physicians, add value to open disclosure [13]. These benefits were generally accepted by participants in this study but some participants provided skeptical responses. In particular, physicians were cynical of the effect of open disclosure on reducing lawsuits and related costs. According to previous studies, some found that open disclosure does not increase the likelihood of lawsuits from the general public [23–25], although one study showed that a fair number of physicians disagreed with its effects on medical lawsuits [26]. Furthermore, two studies of observational data reported that open disclosure reduced the number of medical lawsuits and their related costs [27, 28]. We need to disseminate the known medical lawsuit-related benefits of open disclosure to physicians to improve their understanding of the situation. However, more research is needed to evaluate the medical lawsuit-related effects of open disclosure in non-western countries because most previous studies were conducted in western countries [4].

### Objectives of open disclosure

Another remarkable issue concerns when open disclosure should take place. Most importantly, there were discrepancies between physicians and the general public on the objectives of open disclosure according to the characteristics of the patient safety incidents. First of all, the participants generally agreed on the need for the open disclosure of medical errors causing severe harm. However, some physicians mentioned that they would not conduct open disclosure for medical errors causing minor harm. Furthermore, some physicians expressed marked negativity toward open disclosure of near misses, similar to the findings of previous studies [5, 29]. In particular, we expected that there would be controversy about whether open disclosure is required for near misses. However, as the participants from the general public and some physicians mentioned in this study, a more persuasive argument is that open disclosure of near misses is necessary for the patient’s future reference and to prevent similar medical errors. Gallagher et al. also reported that knowledge of a near miss could alert patients to what medical errors they should be aware of.

Almost all physicians had negative views on open disclosure of inapparent medical errors. However, we believe that these perceptions were caused by the physicians’ misconception that open disclosure only involves apologies for mistakes. When we divided open disclosure into four key elements, namely, providing explanation, showing sympathy and guaranteeing future investigation, offering an apology, and promising adequate amount of compensation and effort to prevent recurrence, providing explanation and showing sympathy and guaranteeing future investigation were also necessary in inapparent medical error cases. Although most of the general public insisted on an apology only when medical error occurred, incident description and sympathy expression were considered essential elements of open disclosure, even for ambiguous medical errors. Medical professionals, including physicians, therefore need to be educated about the key elements of open disclosure. Furthermore, guidelines for open disclosure according to the type of patient safety incident are required to encourage physicians to more readily conduct open disclosure.

### Methods for carrying out open disclosure

In this study, almost none of the interviewed physicians knew how to successfully conduct open disclosure and some reported bad experiences, namely, that inadequate or imperfect open disclosure either provoked negative responses or turned out to be ineffective. Consequently, many physicians seemed to have acquired certain coping skills and they did not practice open disclosure even in situations requiring open disclosure. On the other hand, the general public felt offended by the inadequate open disclosures and felt neglected in other medical areas as well. Therefore, help is required so that physicians can effectively and appropriately carry out open disclosure.

In order to successfully perform open disclosure, a systematic approach, including the development of education programs and guidelines, should be emphasized rather than an individual approach treating open disclosure as an entirely private matter [30–32]. Etchegaray et al. [31] reported that training and education on open disclosure were correlated with a more positive attitude to open disclosure and its benefits in terms of patient trust. Most physicians interviewed in this study also acknowledged the importance of open disclosure education. In particular, it should be offered in medical school and nursing college to future medical professionals. Considering the fact that clinical performance examination and objective structured clinical examination has recently been highlighted, open disclosure training using standardized patients would be one way to educate medical professionals about it.

### Introduction of open disclosure

In general, a focused improvement in the system and medical environment more effectively improves the level of patient safety than blaming and punishing individuals [33]. This is also true in open disclosure and it is difficult for individual medical professionals to conduct open disclosure alone. Furthermore, it is nearly impossible for them to carry out open disclosure without hospital approval. The hierarchical nature of hospital culture, involving apprenticeship and the traditional “blame culture”, was also indicated as a factor further impeding open disclosure. In particular, prior preparation, including organization of a dedicated team and hiring experts for open disclosure, was suggested by our study participants, because single-handed open disclosure is difficult. Indeed, guidelines or standards for open disclosure have been developed to facilitate communication on patient safety incidents between medical professionals and patients in several western countries [8–11]. For example, the guidelines from the Canadian Patient Safety Institute recommend that hospitals organize a team for open disclosure, assign a role to the team members, and create a checklist for open disclosure [8]. However, open disclosure guidelines need to consider the medical culture in each country.

The participants in this study had both positive and negative opinions on apology laws to protect open disclosure. In fact, although western countries such as the United States and Canada have enacted such apology laws, there are no examples of such legislature in non-western countries [3]. Therefore, we believe that reviews of apology laws and evaluations of their feasibility should be first performed in non-western countries with different legal systems, including Korea. However, despite the probability of weakening a patient’s ability to prove medical malpractice in court, most participants from the general public still supported apology laws in this study, indicating a general acceptability of such laws. Although apology laws are divided into full apology laws and partial apology laws according to the range of protection [34], we did not distinguish between them in this study and we did not evaluate perceptions about different components of apology laws in any detail. A future study will be required in this regard.

### Limitations

This study had some limitations of note. First, we did not recruit primary care physicians. Furthermore, other medical professionals such as nurses and allied health professionals, were not included. Further research that includes these types of medical professionals is needed to confirm and generalize the findings of this study. In particular, it would be meaningful to determine the attitude of nurses toward open disclosure as they are typically at the “sharp end” of patient care. An additional limitation was that we only recruited members of the general public with a college diploma. Although it seems that there has been no research on differences in perception regarding open disclosure according to education level, further research that includes a wider sample of the general public is needed to determine the general perception regarding open disclosure. Lastly, we cannot exclude the possibility that the IDI participants and FGD physician participants were not completely open with the researchers and tried to ingratiate themselves with the interviewer in terms of reflexivity.

## Conclusions

Both physicians and the general public in Korea acknowledge the need for open disclosure of patient safety incidents. They suggest that open disclosure has its own value in terms of patient safety and in promoting patient-centered care and they generally accepted the benefits of open disclosure. However, it is difficult for individual medical professionals to conduct open disclosure without support. Guidelines for open disclosure according to the type of patient safety incident are required to encourage physicians to more readily conduct open disclosure. Furthermore, hospitals need to consider organizing a dedicated team and hiring experts for open disclosure.

## Additional file

Additional file 1: Guidelines for the in-depth interviews and focus group discussions including full descriptions of hypothetical cases. (DOCX 20 kb)

## Abbreviations

FGDs: Focus group discussions
IDIs: In-depth interviews
