# Supplementary material for: Perceptions of the general public and physicians regarding open disclosure in Korea: a qualitative study
Source: BMC Med Ethics. 2016 Aug 20;17:50. doi: 10.1186/s12910-016-0134-0 (PMC4992204; doi:10.1186/s12910-016-0134-0)
Supplement: Additional file 1: — Guidelines for the in-depth interviews and focus group discussions including full descriptions of hypothetical cases. (DOCX 20 kb) [file 12910_2016_134_MOESM1_ESM.docx]

**Additional file 1.** Guidelines for the in-depth interviews and focus group discussions

|  | Main questions | |
| --- | --- | --- |
| Icebreaking | • Introduce the discussion and explain its purpose  • Introduce discussion proceedings  ⦁ Introduce participants | |
| Views on open disclosure | Awareness and experience of open disclosure | • “Have you ever heard of open disclosure? Or have you ever had experience with open disclosure?” |
|  | The “5W1H (five Ws and one H)” for open disclosure using four hypothetical cases | • “In the following cases, do you think open disclosure should be performed? Do you think it is necessary?”  • “Reflecting on the definition of open disclosure mentioned in the following case, which of the processes of open disclosure would you choose as a top priority?”  • “If open disclosure were to be performed in the following case, who would be the most appropriate person to perform it?  • “If open disclosure were to be performed in the following case, who would be the most appropriate target?”  • “In the following case, when should open disclosure be performed?”  • “In the following case, where do you think open disclosure should be performed?”  • “In the following case, how should open disclosure be practiced? How would you define sincerity? How can we ascertain his/her sincerity?” |
|  | Verification of obstacles to open disclosure | • “As we have seen in the given case (fifth hypothetical case), it is not easy to perform open disclosure. Some within the medical field point out the difficulty of open disclosure in the lawsuit-prone Korean medical environment. How do you feel about this?”  • “Are there any other obstacles to open disclosure?”  • “The US legal system adopted an apology law to promote open disclosure. What do you think about this?”  • “In contrast, about 10 federal states in the US not only guarantee implementation of open disclosure, but also took a step further to make reporting of adverse event occurrence compulsory. What are your opinions on mandating open disclosure by law, in other words, open disclosure legislation?”  • “The Canadian Patient Safety Institute developed guidelines on open disclosure and recommends its application. Take a look at the guidelines yourself and please share your thoughts on them.” |
| Closing | ⦁ Further questions regarding the focus group discussion  ⦁ Any other questions  ⦁ Conclusion of the focus group discussion | |

**Hypothetical cases**

| Hypothetical case | Description |
| --- | --- |
| 1) Inapparent medical error causing minor harm | A 62-year-old man was admitted to the hospital for surgical treatment of colon cancer diagnosed by colonoscopy. Two days after admission, the treating physician, *Surgeon Na*, successfully performed laparoscopic colon resection. However, the patient presented high fever and tenderness at the surgical site at 3 postoperative days. Imaging tests revealed an abscess at the site where the two ends of the remaining bowel were reconnected. The surgeon suspected leakage from the suture site and inserted a catheter for drainage. The patient recovered without reoperation, but his discharge was delayed by 10 days. |
| 2) Inapparent medical error causing severe harm | A 62-year-old man was admitted to the hospital for surgical treatment of colon cancer diagnosed by colonoscopy. Two days after admission, the treating physician, *Surgeon Na*, successfully performed laparoscopic colon resection. However, the patient presented high fever and tenderness at the surgical site at 3 postoperative days. Imaging tests revealed an abscess at the site where the two ends of the remaining bowel were reconnected. The surgeon suspected leakage from the suture site and performed a colostomy as an emergency procedure. However, the patient was transferred to the intensive care unit due to sepsis and died 7 days after the reoperation due to septic shock. |
| 3) Apparent medical error causing minor harm | A 50-year-old woman was admitted to the hospital with pneumonia. The patient had a past episode of penicillin-related anaphylactic shock. The treating physician, *Medical Kim*, failed to take the medical history into account and prescribed cephalosporin antibiotics. Fortunately, the patient had no other adverse experience than a mild skin rash. She was discharged after 10 days of hospitalization and making a full recovery. |
| 4) Apparent medical error causing severe harm | A 50-year-old woman was admitted to the hospital with pneumonia. The patient had a past episode of penicillin-related anaphylactic shock. The treating physician, *Medical Kim*, failed to take the medical history into account and prescribed cephalosporin antibiotics. As expected, the patient presented with dyspnea, seizure, decreased mentality, and hypotension due to an anaphylactic reaction. Doctor Kim transferred the patient to the ICU and provided intensive care. Luckily, the patient survived, but hypoxic-ischemic injury left her with permanent brain damage. |
| 5) Failure of open disclosure in the case of apparent medical error causing severe harm | A 6-year-old boy was admitted to the hospital for chemotherapy to treat leukemia. On the first day of admission, the resident in charge, *Pediatrics Lee*, prepared cytarabine and vincristine for administration in the injection room. However, Doctor Lee got confused and injected vincristine intrathecally, when it should have been intravenously administered. Doctor Lee was unaware of the error until the patient complained of headache and buttock pain 6 hours after the injection. Analgesics did not ameliorate the pain and paralysis of all four extremities followed. The patient was transferred to the ICU due to development of renal failure. In the end, the patient died 7 days after the erroneous drug administration. Doctor Lee decided to openly discuss this incident and consulted her colleagues, supervisors, and legal department of the hospital on the matter. However, none of her consultants supported open disclosure. As a result, Doctor Lee did not perform open disclosure. |
